# Towards a reliable assessment of Asian elephant population parameters: the application of photographic spatial capture–recapture sampling in a priority floodplain ecosystem

**DOI:** 10.1038/s41598-019-44795-y

**Published:** 2019-06-12

**Authors:** Varun R. Goswami, Mahendra K. Yadava, Divya Vasudev, Parvathi K. Prasad, Pragyan Sharma, Devcharan Jathanna

**Affiliations:** 1 Wildlife Conservation Society – India, Bangalore, 560097 Karnataka India; 2 Centre for Wildlife Studies, Bangalore, 560097 Karnataka India; 3 Conservation Initiatives, Guwahati, 781022 Assam India; 4 Department of Environment and Forest, Govt. of Assam, Guwahati, 781037 Assam India

**Keywords:** Population dynamics, Conservation biology

## Abstract

The hitherto difficult task of reliably estimating populations of wide-ranging megafauna has been enabled by advances in capture–recapture methodology. Here we combine photographic sampling with a Bayesian spatially-explicit capture–recapture (SCR) model to estimate population parameters for the endangered Asian elephant *Elephas maximus* in the productive floodplain ecosystem of Kaziranga National Park, India. Posterior density estimates of herd-living adult females and sub-adult males and females (herd-adults) was 0.68 elephants/km^2^ (95% Credible Intervals, CrI = 0.56−0.81) while that of adult males was 0.24 elephants/km^2^ (95% CrI = 0.18−0.30), with posterior density estimates highlighting spatial heterogeneity in elephant distribution. Estimates of the space-usage parameter suggested that herd-adults (${\hat{\sigma }}_{HA}$ = 5.91 km, 95% CrI = 5.18–6.81) moved around considerably more than adult males (${\hat{\sigma }}_{AM}$ = 3.64 km, 95% CrI = 3.09–4.34). Based on elephant movement and age–sex composition, we derived the population that contributed individuals sampled in Kaziranga to be 908 herd-adults, 228 adult males and 610 young (density = 0.46 young/km^2^, SD = 0.06). Our study demonstrates how SCR is suited to estimating geographically open populations, characterising spatial heterogeneity in fine-scale density, and facilitating reliable monitoring to assess population status and dynamics for science and conservation.

## Introduction

Reliable monitoring is the cornerstone of informed conservation decision making—it provides insights into departures of a system from its desired state, and allows periodic evaluation of the influence of disturbances or perturbations as well as the efficacy of management actions^1,2^. The focus of monitoring programs on population size or density intuitively stems from the important influence these state variables have on a number of demographic and behavioural attributes^3–6^ and thereby long-term population viability. Temporal variation in abundance or density is in turn a function of the underlying vital rates of survival, recruitment and movement, and the estimation of these demographic parameters is a concurrent priority in understanding the drivers of population change^2^. Indeed, unbiased and precise estimation of state variables and associated vital rates transcends applied research, and lays the foundation for the pursuit of several core questions in ecology. Consider, for example, those that pertain to understanding the dynamics and regulation of animal populations^7,8^.

Efforts to monitor populations of species that occur at low densities, occupy dense habitat and range widely are faced with various practical and technical challenges^9–11^. The large spatial scales that are typically involved in monitoring wide-ranging species pose a constraint that can lead to the collection of data that are inadequate or not sufficiently representative of the spatial variation in the distribution of such species. In addition, it is nearly impossible to detect species perfectly in most natural systems. Imperfect detection of species, spatiotemporal variation in detection probability, and inadequate or unrepresentative spatial sampling are primary factors that lead to biased estimates of population size or density^2^, thereby misrepresenting population status and potentially misleading conservation and management decisions. A case in point is that of the endangered and wide-ranging Asian elephant *Elephas maximus*. For the species, a population estimate of 30,000–50,000 wild elephants continues to be cited even when it was recognised over a decade ago that these frequently repeated numbers are educated guesses at best, and not reflective of the multiplying anthropogenic pressures faced by elephants across Asia^12^. Given that elephants worldwide are highly threatened by continuing habitat loss and degradation, staggering spurts in poaching for ivory, and increasing conflict with people^13^, reliable monitoring of their populations is a key priority for conservation policy and action.

The use of visual detections along line transects to estimate population density under a distance sampling framework^9^, and capture–recapture methods involving photograph- or DNA-based identities^14–17^, are now well recognized as reliable approaches to monitor Asian elephant populations. The fact that individual adult elephants can be identified based on their morphological features (e.g., ear lobe shape, tusk orientation, tail length), and can thus be sampled photographically, has particularly enabled the use of capture–recapture models for elephants in both Asia and Africa^14,16–18^. Capture–recapture models have indeed grown tremendously in power and flexibility^19,20^ and a major advantage associated with this framework is that it allows the estimation of both population size, as well as vital rates over time^4,21^.

Conventionally, the use of capture–recapture models to estimate animal density has faced two key constraints: (a) spatially defining an effective sampled area corresponding to the estimated population size *N*; and (b) heterogeneity in capture probabilities induced by variation in exposure of different individuals to sampling^22^. Spatially-explicit capture–recapture (SCR) models address these constraints by treating the data—location- and occasion-specific ‘captures’ of individuals—as the result of two different processes. (a) The state process, which describes the number and distribution of individuals in a defined area *S*; and (b) the observation processes, pertaining to where and when survey effort was invested, and what type of data each detector could record (e.g. counts of encounters, or binary records of encountered/not-encountered individuals for each individual-detector-occasion combination)^20,22,23^. Heterogeneity in capture probabilities is accounted for by the fact that the encounter probability of an individual at a particular sampling location (typically a detector in an array) is determined by the distance of that location from the individual’s activity centre^20^. Recent evidence suggests that SCR is also relatively robust to spatial segregation (or aggregation) of individuals^11^, a characteristic of group-living social animals that could potentially violate the assumption that activity centres are independently distributed over the area *S*^20^. Demographic closure—the assumption that the population is closed to changes from births (or recruitment into a specific age-class) and deaths—can be achieved through a short sampling period relative to the species’ biology. However, it may not always be possible to assume that a population was closed to movement at the time of sampling for wide-ranging species such as elephants. SCR is robust to such geographic non-closure to the extent that it explicitly models animal movement in and out of the sampled area^20^. Indeed, this ability of SCR coupled with the fact that it can account for much of the heterogeneity in capture probability across individuals, makes it a particularly appealing and appropriate approach to reliably assessing populations of wide-ranging species.

**Figure 1:**
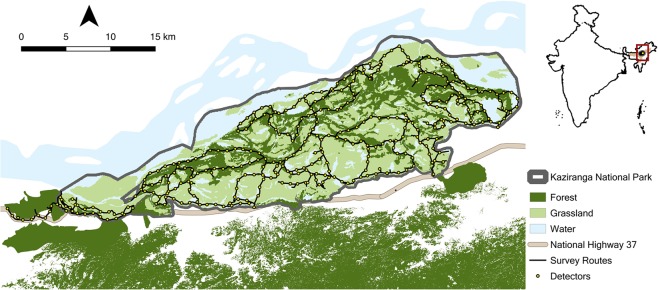
Map of Kaziranga National Park showing major habitat types, water bodies, our survey routes along which elephants were systematically photographed, and detectors to which nearest elephant observations were aggregated. The Brahmaputra River forms a natural boundary for Kaziranga on the north, while adjoining forests, including the highlands of Karbi Anglong to the south, provide additional habitat and refuge for wildlife during periodic floods that inundate large parts of the park.

**Figure 2:**
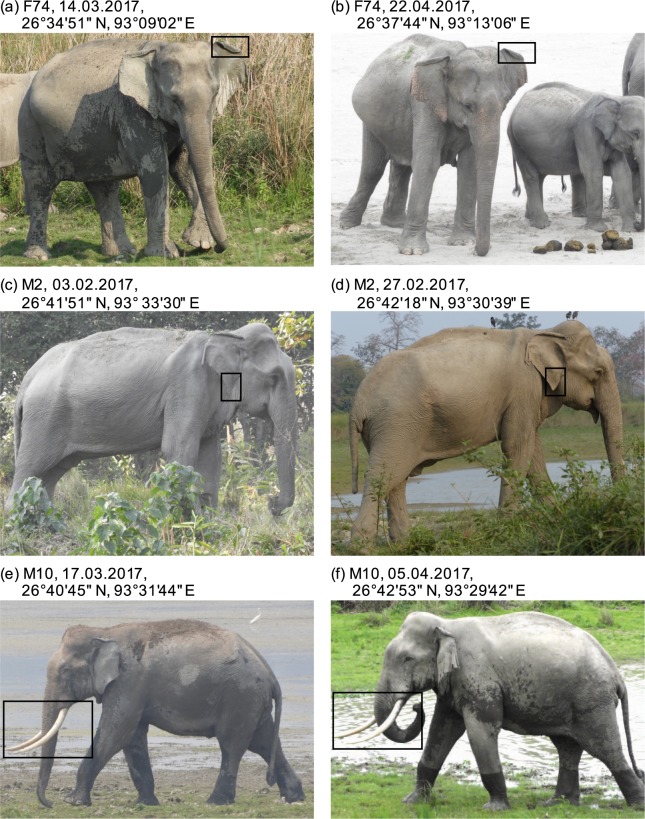
Examples of photographic recaptures of (**a**,**b**) an adult female, (**c**,**d**) a tuskless adult male, and (**e**,**f**) a tusked-adult male. Individual identity numbers, and date and location of the photo-captures are provided. We used a combination of morphological traits to identify individuals. Here, rectangular boxes highlight ear fold (**a**,**b**), shape of the ear lobe (**c**,**d**) and tusk length and angle (**e**,**f**) as examples of morphological traits used.

We present findings from a study where photographic data on individual Asian elephants, systematically collected through replicated surveys across a well-connected road network, are combined with SCR models to estimate population parameters for the species. Our surveys are from Kaziranga National Park (henceforth, Kaziranga), a productive floodplain ecosystem lying on the southern banks of the Brahmaputra River in the state of Assam, Northeast India (Figs 1 and 2), that is known to support a large number of Asian elephants^24^. Previous applications of photographic capture–recapture methods to Asian elephants have either focused on the adult male^14,18^ or the adult female^17^ segment of the elephant population. We build on these efforts to develop field protocols and a survey design that allows the sampling of the entire population in a manner that permits the use of SCR models for analyses. Given that Asian elephant social structure is characterized by female-bonded groups with which largely solitary adult males temporarily associate^25^, we treated (a) the herd-living population of adult females and sub-adult males and females (henceforth, ‘herd-adults’); and (b) the solitary population of adult males, separately for our analyses. We individually identify photo-captured elephants using morphological characteristics. We then use this unambiguous individual identification in an SCR modelling framework to estimate herd-adult and adult male elephant abundance and movement parameters, and map fine-scale elephant densities across Kaziranga. Our findings set the stage for reliable longer-term assessments of ecological factors that underlie spatial heterogeneity in elephant density as well as the influence of different vital rates on elephant population dynamics. In so doing, we demonstrate the feasibility of a robust, spatially-explicit approach to elephant population monitoring across habitats and continents.

## Results

### Effort and individual identification

We conducted photographic surveys for elephants in Kaziranga over 64 sampling occasions, where each occasion spanned a calendar day of effort. During this period, we drove approximately 3300 km, and photographed 890 adult, sub-adult and juvenile elephant sightings. Although only adult Asian elephants have been catalogued previously for capture–recapture analyses^14,17,18^, we successfully documented sub-adult individuals as well. In total, our photographic database included 486 entries of adult and sub-adult females and 234 entries of males. Of these, the number of unique elephants—after disregarding entries where the associated photographic information was not sufficiently complete to assign unambiguous individual identities—comprised 210 adult females, 23 sub-adult females, 88 adult males and 28 sub-adult males. The 88 adult males comprised 48 tuskers and 40 without tusks (*makhnas*), giving an adult tusked to tuskless male ratio of 1.2:1.

### Population estimation

**Figure 3:**
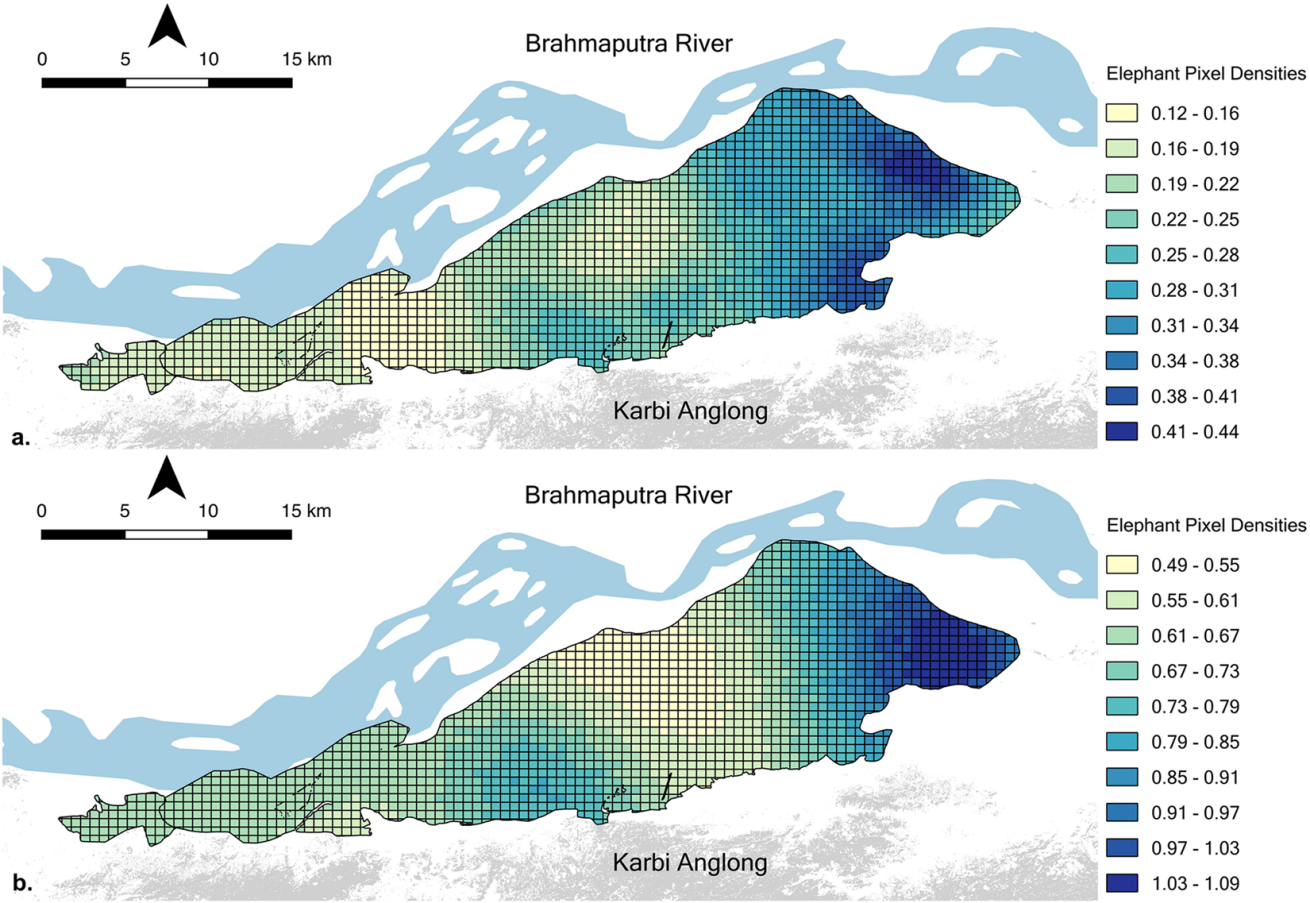
Spatial variation in pixel-specific densities of (**a**) herd-adult and (**b**) adult male elephants within the sampled area, expressed as number of individuals per km^2^.

For herd-adults, Bayesian *P*-value for the SCR model was 0.55, suggesting a good fit, well away from the extremities (<0.15 and >0.85)^10^. The Markov chains indicated convergence, as inferred through visual assessment of the MCMC chains (Figs S2 and S4), and the Gelman–Rubin $\hat{R}$^26^ (Table 1). Geweke *z*-scores for individual chains were between −1.4 and +1.12. The density of herd-adults $({\hat{D}}_{HA})$ in Kaziranga was estimated to be 0.68 individuals/km^2^ (95% Credible Intervals; CrI = 0.56–0.81, Table 1). Fine-scale posterior density estimates ranged from 0.49 herd-adults/km^2^ to 1.09 herd-adults/km^2^, clearly establishing spatial heterogeneity in the distribution of elephant herds in Kaziranga (Figs 3 and S2). This is supported by the estimate of the ‘space-usage’ (or ‘range’) parameter sigma (${\hat{\sigma }}_{HA}$ = 5.91 km, 95% CrI = 5.18–6.81), suggesting that herds moved around considerably about their activity centres. Thus, herd-adults photo-captured in Kaziranga likely included individuals with activity centres both within the park as well as in adjoining habitats to the south, which we denote as the population of herd-adults that contributed individuals that were sampled in Kaziranga. We estimated this population of herd-adults by first computing a buffer around Kaziranga that coincided with the spatial extent of this population; animals with activity centres outside of this buffer had <0.05 probability of moving into Kaziranga, based on estimated *σ*. The radius *r*_0.95_ of this buffer was calculated as *r*_0.95_ = ${\hat{\sigma }}_{HA}\sqrt{5.99}$ of 14.5 km, following Royle *et al*.^20^. We considered habitats within this 14.5 km buffer around Kaziranga (Fig. S1), along with the sampled area of 388 km^2^ in Kaziranga, to obtain an area *A*_0.95 HA_ = 1316 km^2^. We derived the number of herd-adults in the population exposed to sampling to be 908 individuals by summing mean pixel densities within this area *A*_0.95 HA_.

**Table 1:** Posterior mean, 95% Credible Intervals (CrI) and Gelman–Rubin $\hat{R}$ of population parameters estimated for elephants in Kaziranga by the Bayesian SCR model.

Parameter	Herd-adults			Adult males			Definition
	Posterior Mean	Posterior 95% CrI	$\hat{R}$	Posterior Mean	Posterior 95% CrI	$\hat{R}$	
$\sigma$	5.91 km	5.18–6.81	1.011	3.64 km	3.09–4.34	1.000	Scale parameter of a half-normal distribution that represents space-use of individuals about their activity centres and thereby determines the rate of decline in detection probability with increasing distance from the activity centre
${\lambda }_{0}$	0.0009	0.0007–0.0011	1.002	0.002	0.001–0.003	1.000	Basal encounter rate of elephants, or detection probability when activity centres exactly coincide with the trap location
$\psi$	0.80	0.65–0.95	1.009	0.47	0.36–0.61	1.004	Proportion of the maximum possible elephants in the state-space *S*, as provided through data augmentation, that represents the true population
*N*_*super*_	1485	1219–1770	1.002	515	398–661	1.004	Total number of elephants in the state-space *S*
*D*	0.68	0.56–0.81		0.24	0.18–0.30		Estimated elephant density (individuals per km^2^)

Bayesian *P*-value for the SCR model used to estimate the adult male population was 0.60, again indicating a good fit. The Geweke diagnostic^27^ for this model suggested *z*-scores between −0.94 and +1.07 for all parameters. This, along with the Gelman–Rubin $\hat{R}$ (Table 1), supported our visual confirmation of model convergence (Fig. S4). The estimated density of adult males $({\hat{D}}_{AM})$ in Kaziranga was 0.24 individuals/km^2^ (95% CrI = 0.18–0.30, Table 1), with pixel-level posterior density estimates ranging between 0.12 and 0.44 adult males/km^2^. Like herd-living elephants, adult males showed heterogeneity in spatial distribution within Kaziranga (Fig. 3), but they were considerably more localised about their activity centres during the sampling period (${\hat{\sigma }}_{AM}=3.64\,{\rm{km}}$, 95% CrI = 3.09−4.34, Fig. S3). The area corresponding to 95% movement outcomes for the sampled population of adult males during the survey period was computed based on a 95% radius *r*_0.95_ = ${\hat{\sigma }}_{AM}\sqrt{5.99}$ of 8.9 km (Fig. S1) to be *A*_0.95 AM_ = 951 km^2^, accounting for a derived abundance of 228 adult male elephants.

### Age–sex composition

The cumulative numbers of elephants of different age–sex classes that we encountered during the survey is provided in Table 2. The adult male:female ratio based on these counts was 1:2.8. Based on observed age–sex class ratios, we derived the number of juvenile and young elephants that are part of herds in the population corresponding to the area of 1316 km^2^ (*A*_0.95 HA_) as 610 individuals at a density of 0.46 (SD = 0.06) individuals/km^2^.

**Table 2:** Elephant group composition in Kaziranga based on cumulative counts of individuals observed under different age–sex classes.

	Adult males	Herd-adults			Juveniles	Young	Total
		Adult females	Sub-adult females	Sub-adult males			
Number encountered	190	433	61	37	207	153	1081
Proportion in population (SD)	0.18 (0.04)	0.40 (0.04)	0.06 (0.02)	0.03 (0.03)	0.19 (0.03)	0.14 (0.02)	
Density per km^2^	0.24 (95% CrI = 0.18–0.30)^a^	0.68 (95% CrI = 0.56–0.81)^a^			0.46 (0.06 SD)^b^		1.39 (0.15 SD)^b^

^a^Estimated by the SCR model.

^b^Derived based on SCR estimates and observed proportions of age–sex classes.

## Discussion

The growth of capture–recapture methodology over the years has made a substantial contribution to our ability to study and monitor populations of wildlife species across the world^10,28–31^. While the approach is well suited to studies where individuals can be physically tagged^4,31^ or distinguished through natural markings^10,28^, it is increasingly also being applied to contexts where, for example, DNA-based markers allow individual identification^11,32^. This, coupled with the statistical rigour, sophistication and flexibility of capture–recapture models^2,19,20^, makes for a powerful population estimation method for a variety of systems and taxa. There have been multiple calls for the adoption of reliable methods, grounded in estimation theory, for population estimation of Asian elephants^9,12,15,24^. Here, we build on previous efforts that used photographic capture–recapture to estimate adult male^14,18^ and adult female Asian elephant populations^17^, to successfully estimate populations of both adult male and herd-living elephants in Kaziranga, India. In so doing, we unambiguously demonstrate the suitability of capture–recapture methods for reliable monitoring of Asian elephant populations.

Quite significantly, the advancement of capture–recapture to include SCR models has opened up avenues for the examination of populations in relation to space, and thus, variation that is often inherent across it. While SCR affords other important advantages, such as the ability to identify an effective sampled area and account for heterogeneity in capture probabilities^20^, this facet of SCR to allow a visual and analytical assessment of fine-scale spatial variation in species densities, creates exciting opportunities to investigate species–habitat relationships. Moreover, the identification of ‘hotspots’ of local density speaks directly to the management and conservation of focal species. Our results for example demonstrate that the highest densities for both herd-adults and adult male elephants in Kaziranga during the study period occurred in the eastern-most parts of the park (Fig. 3). These parts of Kaziranga have the largest water bodies (Fig. 1), interspersed in more wooded habitat characterised by thickets of cane, primarily of the *Calamus* genus. Thus, our findings likely relate to spatial variation in the availability of resources for elephants, including forage, water and shade. Our results potentially coincide with field observations of wildlife managers in Kaziranga that suggest a possible concentration of elephants towards the east during the February–April period, and the gradual westward movement of elephants thereafter before they ascend to the highlands during the periodic floods of June–September. Although we estimated different detection probabilities for herd adults and adult males, we note that we did not also model these probabilities as a function of factors such as vegetation structure. More detailed assessments of elephant–habitat relationships would need to separate the influence of vegetation characteristics on the process by which elephants are observed (i.e., detection probabilities) from how they determine fine-scale elephant densities.

Our findings demonstrate greater space-use and movement of herd-adults about their activity centres as compared to adult male elephants. Asian elephants are sexually dimorphic, and metabolic needs associated with a larger body size for adult males—coupled with a polygynous mating system—leads to the expectation that adult male elephants should range more widely than herds^33,34^. However, existing literature on Asian elephant ranging behaviour does not report consistent differences between the sexes^35^. Larger male home ranges are often associated with individuals in a state of musth—a temporary period of heightened sexuality and associated physiological, hormonal and behavioural changes in male elephants^36^—as elephants in musth are likely to move about more extensively in search of mates. In our study, only 13 of the 88 adult males we identified were in musth during the sampling period, thereby indicating a relatively small influence of musth on adult male space use. We generally expect home ranges of both herds and adult males to be relatively small in a resource-rich habitat such as Kaziranga. However, given the productivity of the ecosystem, spatial segregation (of herds) that eases intra-specific competition and allows an ‘ecological release’ from strict matriarchal hierarchies, is likely to be favoured^37^. Finally, it is possible that adult males are more localised in their movement in a given season, but demonstrate greater shifts in activity centres than herds with change in season and associated resource availability. Longer-term observations of identified elephants from our system and others could provide greater insight into this and other mechanisms that underlie sexual variation in space-use in Asian elephants.

The SCR model we used estimated elephant densities of 0.68 (95% CrI = 0.56–0.81) herd-adults/km^2^ and 0.24 (95% CrI = 0.18–0.30) adult males/km^2^ for Kaziranga National Park. We combined these estimates with proportions of age–sex classes encountered during the survey to derive the density of juvenile and young elephants to be 0.46 (SD = 0.06) individuals/km^2^ and a total elephant density of 1.39 (SD = 0.15) elephants/km^2^. Our observations point to ratios of 1:2.8 adult males to adult females and 1.2:1 adult tusked to tuskless males, the former ratio being consistent with adult sex ratios of 1:2.3 males to females reported by the Assam Forest Department staff of Kaziranga National Park from surveys conducted in 2005 and 2008^38^. Our population estimates, combined with observed adult sex ratios and the proportion of tusked adult males, reinforce the view that Kaziranga supports a healthy, well-protected elephant source population that lacks the type of skews in adult composition evident in Asian elephant populations elsewhere^25^.

Importantly, however, our *σ* estimates of 5.91 km (95% CrI = 5.18–6.81) and 3.64 km (95% CrI = 3.09–4.34) for herd-adults and adult males, respectively, suggest that habitats outside Kaziranga represent a critical part of the larger landscape that supports thriving populations of elephants and other wildlife. Thus, our findings reiterate the importance of conserving elephant habitats in Kaziranga and Karbi Anglong, as well as potential movement conduits between them, as complementary parts of a nearly continuous landscape that together maintain functionality of the ecosystem as a whole. Extending the monitoring approach described here to the larger landscape, combined with multi-state capture–recapture models, would allow the estimation of movement probabilities in and out of Kaziranga.

We estimated the population of herd-adults that contributed to individuals being photo-captured in Kaziranga to include 908 elephants. Similarly, we estimated the adult male population that contributed to sampled individuals to comprise 228 elephants. While these estimates underscore the overall importance of the Kaziranga–Karbi Anglong landscape as a priority site for long-term conservation of Asian elephants, we also recommend caution in how these estimates are interpreted. Our photographic surveys were restricted to Kaziranga and therefore fine-scale density estimates for within Kaziranga (Fig. 3) are the most robust. As we move further away from the sampled area, there is less information from actual detections of elephants on spatial heterogeneity in predicted fine-scale density. At sufficiently large distances the predicted fine-scale densities reflect the average density across the state-space. It is important to mention here that the hills of Karbi Anglong have by-and-large been difficult to access for the purposes of wildlife monitoring or conservation due to socio-political reasons. Anecdotal information suggests that wildlife populations may be subject to various anthropogenic threats in some of those habitats, which could result in lower than expected elephant densities.

We conclude, therefore, by recommending that ongoing efforts to research and conserve wildlife populations in Kaziranga be proactively complemented with similar engagement in Karbi Anglong to the extent feasible. It would be imperative that such efforts utilise and build on what we report here, such that the health of wildlife populations in the landscape, and the impact of various conservation efforts to mitigate the threats they may face, can be reliably monitored. A comprehensive monitoring program of the kind would be critical to informing an adaptive management system that can help sustain one of planet Earth’s most diverse and rich floodplain ecosystems.

## Methods

### Study area

Kaziranga, first proposed as a reserved forest in 1905, was notified as a National Park in 1974, covering an area of 429.93 km^2^ south of the Brahmaputra River^38^. However, subsequent flood dynamics and erosion by the Brahmaputra on its south bank, led to a loss of 48.89 km^2^ and a gain of 7.44 km^2^ in Kaziranga’s land area^38^. Our sampled area thus spanned a 388-km^2^ expanse of Kaziranga National Park (26°34′–26°45′N, 93°00′–93°35′E), coinciding with the current coverage of what was first notified as a National Park, prior to subsequent additions to the park area through various government notifications^39^. Primarily comprising grassland, mixed deciduous and semi-evergreen forests, the productive habitat of Kaziranga—a UNESCO natural World Heritage Site since 1985—is shaped by the annual flooding of the Brahmaputra. To the south of Kaziranga are the hills of Karbi Anglong, comprised mainly of mixed deciduous, semi-evergreen and evergreen forests. In addition to elephants, Kaziranga supports the largest extant population of the greater one-horned rhinoceros *Rhinoceros unicornis*, one of the highest densities of tigers *Panthera tigris*, and substantial populations of the Asiatic water buffalo *Bubalus arnee*, the Indian barasingha or swamp deer *Rucervus duvaucelii* and hog deer *Axis porcinus*^24,39–41^. Indeed, the unique assemblage of sympatric herbivores in Kaziranga is amongst the richest in diversity and density across South Asia^38,41^.

Each year, inundation of Kaziranga during the monsoon season (June–September) necessitates the movement of its animals, including elephants, towards the hills of Karbi Anglong to the south. The hills, therefore, are integral to the survival of elephants in the landscape and form a part of the Kaziranga–Karbi Anglong Elephant Reserve, a priority elephant habitat in Northeast India^24^. Encompassing an area of 3270 km^2^, the Elephant Reserve comprises Protected Areas, Reserve Forests, human land-uses such as tea estates and agricultural lands. While elephants outside Kaziranga come into contact with various anthropogenic threats, the park itself acts as an important and well-protected habitat within the larger Elephant Reserve. The source population in Kaziranga thus holds the key to long-term viability of elephants in the landscape.

### Field data collection

We used a capture–recapture sampling framework to systematically survey Kaziranga and obtain photographic data from direct observations of elephants. We conducted our survey by driving along a predetermined road network of 330 km within Kaziranga, divided into 10 routes for logistical reasons (Fig. 1). The 10 routes together covered the expanse of Kaziranga such that 95% of locations within the study area were ≤1.5 km from the nearest detector, with the maximum distance being 3.8 km. We conducted our survey during the dry season, from 1^st^ February 2017 to 25^th^ April 2017. We covered the entire study area in one sweep over six days, and repeated 10 such sweeps to maximise spatial recaptures of elephants across the study area, but within the 84-day overall survey period to minimise chances of demographic non-closure.

During the survey, we photographed all elephants encountered with the intent of individually identifying them based on previously developed protocols^14,18^. To the extent possible, we obtained a complete *photograph set* of all detected elephants, which included photographs of the front, back, and right and left profiles. For each sighting, we additionally recorded: (a) GPS coordinates of the observer, (b) distance of, and (c) bearing to the individual elephant or centre of the herd. These data enabled us to obtain the geographic location of the elephant or herd. We also recorded (d) date and time of the sighting, (e) herd identity and (f) herd size and composition, for each sighting. For herd composition, we followed methods described earlier^17^ to classify elephants into seven age–sex classes: adult male, adult female, sub-adult male, sub-adult female, juvenile, infant and new-born. For our analyses, we combined infant and new-born into a single age–sex class, denoted as ‘young’.

### Identification of individual elephants

We created a photographic database of elephants using the software FileMaker Pro Advanced (version 14.0.3, FileMaker Inc., Santa Clara, USA). We included in the database, all adult, sub-adult and juvenile elephants for which we were able to clearly determine morphological characteristics necessary for reliable identification.

From these, we identified individual elephants based on morphological traits, primarily ears, tail, and tusks (for males) following earlier studies^14,18^. Each elephant was assigned a unique ID upon ensuring that it was different from all other individuals in the database; this ID was then attached to all subsequent recaptures of the same elephant. For some photo-captures, we were able to only obtain partial identities, or single-sided photographs. To avoid misidentification, we counted the number of photo-identities with ‘left-only’ or ‘right-only’ photographs, and excluded from further analyses the category with fewer records. We thus discarded 31 left-only photo-identities for herd-adults and 11 right-only photo-identities for adult males. Therefore, our identities were unambiguously based on both-side images plus images of the side for which we had more photo-identities.

Based on elephant social structure—corroborated by field observations where we found adult females and sub-adults (both males and females) to occur in herds that were largely independent of solitary adult males—we segregated these data into two sets: (a) herd-adults, comprising adult females and sub-adults, and (b) adult males. We performed subsequent analyses separately on each of these datasets.

### Estimation of population density

We estimated density using SCR, a framework that explicitly accounts for the spatial structure of ecological processes, in this case, the distribution of elephants in space^22,24,25^. SCR models are hierarchical in nature, comprising two components—one describing the underlying ecological process or the distribution and number of animal activity centres across a defined area of space, and the other describing the observation or detection process conditional on the ecological process. The detection model can be viewed as a combination of two distinct processes: (a) movement of an elephant about its activity centre (i.e., its space-usage), and (b) detection of this elephant, conditional on its use of the space near a detector^20^. Accordingly, SCR models quantify individual detection probabilities as a function of the distance between animal activity centres and detectors, and we used a half-normal detection function to model detection probability. Detection probabilities are thus described in terms of two parameters—a scale parameter *σ* that describes space usage around the animal activity centre and thus determines the rate of decline of detection probability as a function of the distance between the activity centre and the detector, and a basal encounter rate *λ*_0_ which represents the expected encounter rate when the distance between the animal activity centre and detector is 0 (Table 1). We assumed no behavioural response to sampling. We implemented this Bayesian SCR model in program SPACECAP^42^ to separately estimate the herd-adult and adult-male population densities.

We defined a state-space *S* of 5639.64 km^2^, inclusive of the study area and a 25-km buffer (Fig. S1). The size of the state-space was chosen such that, for the duration of the survey, animals at the edge of the state-space had negligible probability of being captured within our sampled area. We overlaid a grid with pixels of size 0.314 km^2^ over the state-space; the pixel size was chosen to allow sufficient spatial resolution in assessing potential locations of animal activity centres. We classified the area within the state-space as habitat or non-habitat: forest areas—both protected and unprotected—were categorized as habitat, while non-habitat, comprising human habitation, roads, tea plantations, agricultural areas and fallow lands, were excluded. In addition, since the Brahmaputra acts as a natural barrier to elephant movement, we classified the river and the region to its north as non-habitat. In all, an area of 2192.52 km^2^ was marked as habitat within the state-space *S*. The centroid of each pixel marked as ‘habitat’ represented potential animal activity centres.

We subdivided each of our 10 sampling routes into 1-km stretches, the centre of which formed our pseudo-detectors. This provided us with a total of 350 pseudo-detectors in our sampled area. These detectors were surveyed over the 64 sampling occasions, and for each occasion, we marked as ‘active’ the detectors that corresponded to the route covered on that day. Since certain detectors were common to multiple routes and were therefore traversed more frequently, such an approach allowed us to account for uneven effort per detector. We aggregated detections of elephants, recorded all along the sampling routes, to the nearest detector^43^. Most detections were within 0.5 km from the detector to which it was aggregated. Around 7.5% were further away from the nearest detector, with only one sighting being >1 km away at a distance of 1.8 km. At this distance of aggregation, which is small relative to the expected space-usage of elephants (and the ${\hat{\sigma }}_{HA}$ and ${\hat{\sigma }}_{AM}$ estimated in our study), SCR is robust to aggregated binary detections of individuals^43^. We combined the spatial and temporal information of animal sightings obtained in the field in a three-dimensional matrix consisting of animal ID, trap location and sampling occasion.

We augmented our data with a large number of individuals with zero-only detection histories, to account for those individuals that may have been present in the study area but remained undetected, to provide a total number of individuals (i.e., detected and augmented) *M*. The estimated *ψ* is the proportion of (detected and augmented) individuals that form the true population within the state-space *S*. The state of *M* individuals is a latent variable *z* denoting whether each individual is a member of the true population within the state-space *S*—where *z*_*i*_ = 1 for individuals that are part of the true population, and *z*_*i*_ = 0 for those that are not. *z* is a Binomial random variable with probability *ψ* and size *M*. The derived population size parameter *N*_*super*_ corresponds to the number of elephant activity centres within the state-space *S* and is the sum of all individuals with state *z*_*i*_ = 1 for each iteration. We augmented the dataset of 88 identified adult male elephants with 1000 individuals, and the dataset of 261 identified herd-adults with 1600 individuals respectively, chosen so that the posterior distribution of *ψ* would not be truncated.

We adopted a Bayesian SCR model using MCMC simulations, implemented in R^44^ using package SPACECAP^42^. We ran three chains with 70,000 iterations each for adult male elephants, and 80,000 iterations each for herd-adults, each with a burn-in of 30,000 and thinning rate of 4, resulting in a posterior sample of 10,000 and 12,500 iterations per chain, respectively. Convergence of the individual chains was assessed using the Geweke diagnostic^27^, and a visual assessment of the MCMC chains; if convergence was not achieved, we evaluated why that was the case, and accordingly modified the burn-in period and/or the number of individuals used for data augmentation. We then assessed convergence across the three chains using the Gelman–Rubin $\hat{R}$ as well as a visual assessment of the overlap of the three chains. We used the package coda^45^ in program R^44^ and RStudio^46^ to extract the Gelman–Rubin $\hat{R}$, and to obtain 95% Credible Intervals (CrI) for all estimated parameters.

In addition to parameter estimates and posterior densities, program SPACECAP^42^ provides individual animal densities at the level of pixels. For each iteration, each pixel is either assigned an activity centre or not. A subset of these activity centres is for those individuals that are part of the true population, that is, where z_i_ = 1. When this subset is averaged across iterations, it provides pixel-level densities. We map pixel (fine-scale) densities averaged across all iterations.

To estimate the population of herd-adults that contributed individuals that were sampled in Kaziranga, we began by identifying the space that corresponded to this population. To achieve this, we (a) computed a 95% radius *r*_0.95_ = $${\hat{\sigma }}_{HA}$$$$\sqrt{5.99}$$ of 14.5 km by treating successive locations of an individual to represent draws from a bivariate normal distribution with standard deviation *σ*, and thereby treating the distance of successive locations from the activity centre as a chi-square distribution with 2 degrees of freedom; and (b) considered habitats within a 14.5 km buffer around Kaziranga along with the sampled area of 388 km^2^ in Kaziranga, to compute an area *A*_0.95 HA_ wherein animals with activity centres outside *A*_0.95 HA_ had <0.05 probability of moving into Kaziranga, based on $${\hat{\sigma }}_{HA}$$^20^. Summed pixel densities for this area provided a derived abundance of herd-adults in the population. Similarly, we computed the area corresponding to 95% movement outcomes for the sampled population of adult males during the survey period (*A*_0.95 AM_) based on their estimated space-use ($${\hat{\sigma }}_{AM}$$), and summed pixel densities for that area to derive the abundance of adult males in the population.

Finally, we used the cumulative counts of all individuals encountered under the different age–sex classes to estimate their proportions in the population (Table 2), averaged over the 10 sweeps of Kaziranga. Following methods described previously^14^, we thereafter multiplied the ratio of the proportion of juveniles and infants, to the proportion of herd-adults, with the estimated herd-adult density $({\hat{D}}_{HA})$ to derive the combined density of juvenile and young elephants in the sampled population. This was added to the estimated herd-adult $({\hat{D}}_{HA})$ and adult male $({\hat{D}}_{AM})$ densities to then obtain the total elephant density for the sampled population^14^. As juvenile and young are part of elephant herds, we also derived their abundance in the population based on the area corresponding to *A*_0.95 HA_.

## Supplementary information

Supplementary Information

## Data Availability

All data generated or analysed during this study are included in this published article (and its Supplementary Information files).
