# Author Correction: Towards a reliable assessment of Asian elephant population parameters: the application of photographic spatial capture–recapture sampling in a priority floodplain ecosystem

**DOI:** 10.1038/s41598-019-51389-1

**Published:** 2019-10-09

**Authors:** Varun R. Goswami, Mahendra K. Yadava, Divya Vasudev, Parvathi K. Prasad, Pragyan Sharma, Devcharan Jathanna

**Affiliations:** 1 Wildlife Conservation Society – India, Bangalore, 560097 Karnataka India; 2 Centre for Wildlife Studies, Bangalore, 560097 Karnataka India; 3 Conservation Initiatives, Guwahati, 781022 Assam India; 4 Department of Environment and Forest, Govt. of Assam, Guwahati, 781037 Assam India

Correction to: *Scientific Reports* 10.1038/s41598-019-44795-y, published online 12 June 2019

In this Article, the legend of Figure 3 is incorrect:

“Spatial variation in pixel-specific densities of (**a**) herd-adult and (**b**) adult male elephants within the sampled area, expressed as number of individuals per km^2^.”

should read:

“Spatial variation in pixel-specific densities of (**a**) adult male and (**b**) herd-adult elephants within the sampled area, expressed as number of individuals per km^2^.”

