# Supplementary material for: Towards a reliable assessment of Asian elephant population parameters: the application of photographic spatial capture–recapture sampling in a priority floodplain ecosystem
Source: Sci Rep. 2019 Jun 12;9:8578. doi: 10.1038/s41598-019-44795-y (PMC6561924; doi:10.1038/s41598-019-44795-y)
Supplement: Supplementary file 1 — Supplementary Information [file 41598_2019_44795_MOESM1_ESM.docx]

**Supplementary Information**

**Towards a reliable assessment of Asian elephant population parameters: the application of photographic spatial capture–recapture sampling in a priority floodplain ecosystem**

Varun R. Goswami, M. K. Yadava, Divya Vasudev, Parvathi K. Prasad, Pragyan Sharma & Devcharan Jathanna

**Figure S1.** Asian elephant habitat outside the study area in Kaziranga that contributed to the SCR-based population estimates. The study area and habitats up to (i) a buffer of 25 km comprised the entire state-space; (ii) a buffer of 14.5 km represented the area corresponding to the population of herd-adults; and (iii) a buffer of 8.9 km supported the estimated population of adult males that contributed to individuals being photo-captured in Kaziranga. Areas in white within the state-space were assumed to be non-habitat in the SCR model.


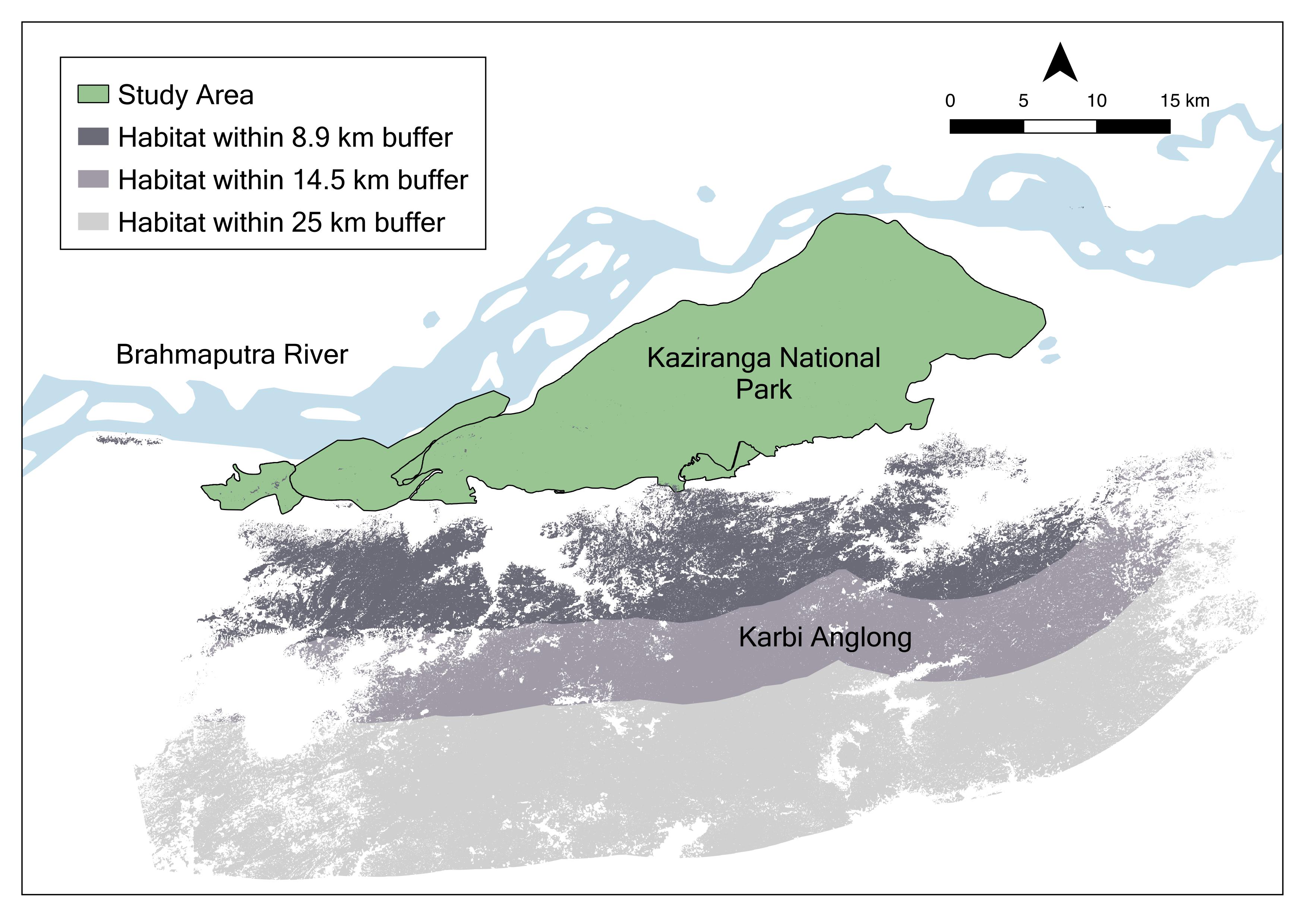


**Figure S2.** Posterior distributions of estimated and derived parameters from the SCR model used for the population of adult female and sub-adult male and female Asian elephants (i.e., herd-adults) in Kaziranga.

| $\sigma$   | $\lambda_{0}$   |
| --- | --- |
| *N_super_*   | $\psi$   |
|  | |

**Figure S3.** MCMC chains (shown in different colours) for estimated and derived parameters from the SCR model used for the population of herd-adult Asian elephants in Kaziranga.

| $\sigma$   | $\lambda_{0}$   |
| --- | --- |
| *N_super_*   | $\psi$   |
|  | |

**Figure S4.** Posterior distributions of estimated and derived parameters from the SCR model used for the population of adult male Asian elephants in Kaziranga.

| $\sigma$   | $\lambda_{0}$   |
| --- | --- |
| *N_super_*   | $\psi$   |
|  | |

**Figure S5.** MCMC chains for estimated and derived parameters from the SCR model used for the population of adult male Asian elephants in Kaziranga.

| $\sigma$   | $\lambda_{0}$   |
| --- | --- |
| *N_super_*   | $\psi$   |
|  | |

**Table S1.** Photographic capture histories for (a) herd-adult and (b) adult male Asian elephants in Kaziranga.

(a) Herd-adults

| Animal ID | Trap location  ID | Sampling occasion |
| --- | --- | --- |
| 1 | 271 | 1 |
| 2 | 184 | 2 |
| 3 | 208 | 18 |
| 4 | 281 | 18 |
| 5 | 271 | 18 |
| 6 | 271 | 18 |
| 6 | 169 | 45 |
| 7 | 272 | 18 |
| 7 | 321 | 30 |
| 8 | 102 | 19 |
| 8 | 34 | 34 |
| 9 | 16 | 58 |
| 9 | 12 | 55 |
| 10 | 135 | 19 |
| 10 | 184 | 51 |
| 11 | 100 | 19 |
| 12 | 100 | 19 |
| 13 | 184 | 2 |
| 14 | 100 | 19 |
| 14 | 267 | 46 |
| 15 | 100 | 19 |
| 15 | 267 | 46 |
| 16 | 100 | 19 |
| 16 | 186 | 29 |
| 17 | 72 | 62 |
| 17 | 190 | 20 |
| 18 | 148 | 20 |
| 19 | 148 | 20 |
| 20 | 184 | 2 |
| 21 | 148 | 20 |
| 21 | 253 | 35 |
| 22 | 184 | 20 |
| 23 | 289 | 20 |
| 24 | 289 | 20 |
| 24 | 268 | 35 |
| 25 | 289 | 20 |
| 25 | 269 | 35 |
| 26 | 289 | 20 |
| 27 | 184 | 2 |
| 28 | 289 | 20 |
| 29 | 289 | 20 |
| 30 | 289 | 20 |
| 31 | 290 | 59 |
| 31 | 290 | 20 |
| 31 | 305 | 35 |
| 31 | 335 | 52 |
| 32 | 290 | 20 |
| 33 | 278 | 20 |
| 34 | 278 | 20 |
| 35 | 261 | 20 |
| 35 | 229 | 51 |
| 36 | 330 | 22 |
| 36 | 305 | 35 |
| 36 | 255 | 43 |
| 37 | 184 | 2 |
| 38 | 330 | 22 |
| 39 | 130 | 25 |
| 40 | 239 | 26 |
| 41 | 239 | 26 |
| 42 | 151 | 28 |
| 43 | 149 | 28 |
| 44 | 149 | 28 |
| 45 | 149 | 28 |
| 46 | 149 | 28 |
| 47 | 184 | 2 |
| 48 | 186 | 29 |
| 49 | 278 | 30 |
| 49 | 266 | 46 |
| 50 | 292 | 30 |
| 51 | 292 | 30 |
| 52 | 292 | 30 |
| 52 | 320 | 30 |
| 53 | 292 | 30 |
| 54 | 319 | 30 |
| 55 | 320 | 30 |
| 56 | 184 | 2 |
| 56 | 317 | 56 |
| 57 | 321 | 30 |
| 58 | 340 | 30 |
| 59 | 341 | 30 |
| 60 | 277 | 31 |
| 61 | 340 | 31 |
| 62 | 169 | 31 |
| 63 | 78 | 32 |
| 63 | 186 | 52 |
| 64 | 216 | 34 |
| 65 | 184 | 2 |
| 66 | 252 | 35 |
| 67 | 253 | 35 |
| 68 | 253 | 35 |
| 69 | 253 | 35 |
| 70 | 305 | 35 |
| 71 | 278 | 35 |
| 72 | 278 | 35 |
| 73 | 278 | 35 |
| 74 | 287 | 35 |
| 75 | 184 | 2 |
| 76 | 268 | 35 |
| 77 | 268 | 35 |
| 78 | 268 | 35 |
| 79 | 289 | 20 |
| 79 | 268 | 35 |
| 80 | 269 | 35 |
| 81 | 42 | 36 |
| 81 | 175 | 48 |
| 82 | 42 | 36 |
| 83 | 320 | 37 |
| 84 | 320 | 37 |
| 85 | 320 | 37 |
| 86 | 340 | 37 |
| 87 | 68 | 38 |
| 87 | 162 | 52 |
| 88 | 68 | 38 |
| 89 | 68 | 38 |
| 90 | 94 | 38 |
| 90 | 186 | 39 |
| 91 | 191 | 40 |
| 92 | 186 | 40 |
| 93 | 271 | 1 |
| 94 | 332 | 40 |
| 95 | 156 | 41 |
| 96 | 156 | 41 |
| 97 | 253 | 42 |
| 98 | 253 | 42 |
| 99 | 254 | 42 |
| 100 | 304 | 42 |
| 101 | 206 | 43 |
| 102 | 191 | 44 |
| 103 | 191 | 44 |
| 104 | 191 | 44 |
| 105 | 266 | 46 |
| 106 | 266 | 46 |
| 107 | 266 | 46 |
| 108 | 267 | 46 |
| 109 | 154 | 47 |
| 110 | 154 | 47 |
| 111 | 191 | 49 |
| 112 | 191 | 49 |
| 113 | 191 | 49 |
| 114 | 191 | 49 |
| 115 | 209 | 49 |
| 116 | 340 | 50 |
| 117 | 319 | 2 |
| 117 | 289 | 20 |
| 118 | 229 | 51 |
| 119 | 229 | 51 |
| 120 | 229 | 51 |
| 121 | 184 | 51 |
| 122 | 290 | 59 |
| 122 | 335 | 52 |
| 122 | 289 | 56 |
| 123 | 167 | 52 |
| 124 | 167 | 52 |
| 125 | 154 | 52 |
| 126 | 334 | 2 |
| 126 | 338 | 18 |
| 126 | 335 | 52 |
| 126 | 289 | 56 |
| 127 | 186 | 52 |
| 128 | 148 | 52 |
| 129 | 118 | 53 |
| 130 | 118 | 53 |
| 131 | 118 | 53 |
| 132 | 154 | 62 |
| 132 | 118 | 53 |
| 133 | 127 | 53 |
| 134 | 12 | 55 |
| 135 | 12 | 55 |
| 136 | 12 | 55 |
| 137 | 334 | 2 |
| 137 | 338 | 18 |
| 137 | 335 | 52 |
| 138 | 317 | 56 |
| 139 | 317 | 56 |
| 140 | 317 | 56 |
| 141 | 317 | 56 |
| 142 | 287 | 56 |
| 143 | 292 | 56 |
| 144 | 289 | 56 |
| 145 | 289 | 56 |
| 146 | 289 | 56 |
| 147 | 334 | 2 |
| 147 | 338 | 18 |
| 147 | 335 | 52 |
| 147 | 289 | 56 |
| 148 | 9 | 58 |
| 149 | 9 | 58 |
| 150 | 9 | 58 |
| 151 | 9 | 58 |
| 152 | 30 | 58 |
| 153 | 30 | 58 |
| 154 | 267 | 59 |
| 155 | 278 | 59 |
| 156 | 278 | 59 |
| 157 | 278 | 59 |
| 158 | 137 | 3 |
| 159 | 278 | 59 |
| 160 | 293 | 59 |
| 161 | 175 | 61 |
| 162 | 175 | 61 |
| 163 | 154 | 62 |
| 164 | 154 | 62 |
| 165 | 72 | 62 |
| 166 | 84 | 62 |
| 167 | 84 | 62 |
| 168 | 84 | 62 |
| 169 | 192 | 3 |
| 169 | 229 | 51 |
| 170 | 94 | 62 |
| 171 | 321 | 63 |
| 172 | 332 | 40 |
| 173 | 152 | 41 |
| 174 | 289 | 20 |
| 175 | 184 | 4 |
| 176 | 271 | 1 |
| 176 | 271 | 8 |
| 177 | 110 | 4 |
| 178 | 190 | 4 |
| 178 | 121 | 19 |
| 179 | 190 | 4 |
| 179 | 289 | 20 |
| 180 | 190 | 4 |
| 180 | 121 | 19 |
| 181 | 321 | 6 |
| 181 | 271 | 35 |
| 182 | 332 | 6 |
| 183 | 16 | 58 |
| 183 | 39 | 7 |
| 183 | 12 | 55 |
| 184 | 84 | 62 |
| 184 | 39 | 7 |
| 184 | 12 | 55 |
| 185 | 337 | 1 |
| 186 | 30 | 58 |
| 186 | 39 | 7 |
| 187 | 30 | 58 |
| 187 | 39 | 7 |
| 188 | 320 | 8 |
| 188 | 267 | 46 |
| 189 | 320 | 8 |
| 189 | 267 | 46 |
| 190 | 321 | 8 |
| 190 | 291 | 30 |
| 190 | 267 | 46 |
| 191 | 272 | 8 |
| 192 | 269 | 8 |
| 192 | 272 | 13 |
| 192 | 275 | 22 |
| 193 | 279 | 1 |
| 194 | 72 | 9 |
| 195 | 72 | 9 |
| 196 | 72 | 9 |
| 196 | 149 | 28 |
| 197 | 72 | 9 |
| 197 | 94 | 14 |
| 198 | 72 | 9 |
| 199 | 72 | 9 |
| 199 | 94 | 14 |
| 200 | 279 | 1 |
| 200 | 289 | 56 |
| 201 | 72 | 9 |
| 201 | 94 | 14 |
| 201 | 175 | 56 |
| 202 | 72 | 9 |
| 202 | 94 | 14 |
| 202 | 175 | 56 |
| 203 | 72 | 9 |
| 203 | 94 | 14 |
| 203 | 175 | 56 |
| 204 | 72 | 9 |
| 205 | 72 | 9 |
| 206 | 72 | 9 |
| 207 | 72 | 62 |
| 207 | 98 | 9 |
| 207 | 121 | 19 |
| 207 | 167 | 52 |
| 208 | 94 | 62 |
| 208 | 98 | 9 |
| 208 | 167 | 52 |
| 209 | 154 | 62 |
| 209 | 98 | 9 |
| 209 | 98 | 9 |
| 209 | 149 | 28 |
| 210 | 98 | 9 |
| 210 | 175 | 48 |
| 211 | 98 | 9 |
| 211 | 121 | 19 |
| 212 | 191 | 58 |
| 212 | 98 | 9 |
| 212 | 121 | 19 |
| 212 | 149 | 28 |
| 213 | 98 | 9 |
| 213 | 121 | 19 |
| 213 | 149 | 28 |
| 213 | 127 | 53 |
| 214 | 98 | 9 |
| 214 | 98 | 9 |
| 214 | 121 | 19 |
| 214 | 149 | 28 |
| 215 | 30 | 58 |
| 215 | 84 | 62 |
| 215 | 83 | 9 |
| 215 | 34 | 34 |
| 215 | 106 | 44 |
| 216 | 83 | 9 |
| 216 | 12 | 55 |
| 217 | 83 | 9 |
| 218 | 98 | 9 |
| 218 | 149 | 28 |
| 219 | 150 | 9 |
| 219 | 190 | 20 |
| 219 | 162 | 52 |
| 220 | 94 | 62 |
| 220 | 33 | 10 |
| 220 | 148 | 20 |
| 220 | 154 | 52 |
| 221 | 33 | 10 |
| 221 | 154 | 52 |
| 222 | 33 | 10 |
| 222 | 148 | 20 |
| 222 | 154 | 52 |
| 223 | 94 | 62 |
| 223 | 33 | 10 |
| 223 | 154 | 52 |
| 224 | 94 | 62 |
| 224 | 33 | 10 |
| 225 | 33 | 10 |
| 225 | 154 | 52 |
| 226 | 258 | 11 |
| 227 | 216 | 12 |
| 228 | 271 | 13 |
| 228 | 271 | 35 |
| 229 | 272 | 13 |
| 229 | 289 | 20 |
| 229 | 320 | 30 |
| 230 | 135 | 14 |
| 231 | 94 | 14 |
| 232 | 72 | 14 |
| 232 | 175 | 41 |
| 233 | 72 | 14 |
| 234 | 317 | 56 |
| 235 | 317 | 56 |
| 236 | 287 | 56 |
| 237 | 127 | 62 |
| 238 | 121 | 19 |
| 239 | 320 | 37 |
| 240 | 106 | 44 |
| 241 | 335 | 52 |
| 241 | 289 | 56 |
| 242 | 118 | 53 |
| 243 | 83 | 9 |
| 243 | 12 | 55 |
| 244 | 317 | 56 |
| 245 | 17 | 58 |
| 246 | 167 | 52 |
| 247 | 84 | 62 |
| 248 | 39 | 7 |
| 248 | 12 | 55 |
| 249 | 184 | 2 |
| 250 | 270 | 18 |
| 250 | 320 | 18 |
| 250 | 289 | 20 |
| 251 | 320 | 18 |
| 252 | 121 | 19 |
| 253 | 289 | 20 |
| 253 | 169 | 31 |
| 254 | 289 | 20 |
| 255 | 289 | 20 |
| 256 | 42 | 36 |
| 257 | 335 | 40 |
| 258 | 30 | 58 |
| 259 | 253 | 42 |
| 260 | 255 | 43 |
| 261 | 94 | 62 |
| 261 | 191 | 44 |

**Total number of sampling occasions: 64*

(b) Adult males

| Animal ID | Trap location  ID | Sampling occasion |
| --- | --- | --- |
| 1 | 291 | 1 |
| 1 | 317 | 1 |
| 2 | 272 | 50 |
| 2 | 312 | 2 |
| 2 | 324 | 59 |
| 2 | 333 | 40 |
| 2 | 335 | 37 |
| 2 | 335 | 40 |
| 3 | 105 | 54 |
| 4 | 12 | 55 |
| 5 | 160 | 56 |
| 6 | 318 | 56 |
| 7 | 289 | 56 |
| 8 | 72 | 53 |
| 8 | 94 | 14 |
| 9 | 320 | 8 |
| 9 | 331 | 2 |
| 9 | 335 | 37 |
| 10 | 186 | 58 |
| 11 | 184 | 58 |
| 11 | 262 | 46 |
| 12 | 190 | 60 |
| 12 | 191 | 58 |
| 12 | 191 | 60 |
| 12 | 191 | 61 |
| 12 | 191 | 62 |
| 13 | 168 | 61 |
| 14 | 154 | 62 |
| 15 | 119 | 62 |
| 16 | 155 | 3 |
| 17 | 184 | 55 |
| 18 | 278 | 42 |
| 18 | 289 | 56 |
| 18 | 321 | 8 |
| 18 | 332 | 6 |
| 19 | 49 | 7 |
| 20 | 72 | 9 |
| 20 | 118 | 14 |
| 21 | 72 | 9 |
| 21 | 148 | 20 |
| 22 | 270 | 20 |
| 22 | 270 | 22 |
| 22 | 272 | 37 |
| 22 | 313 | 46 |
| 22 | 326 | 1 |
| 22 | 332 | 2 |
| 23 | 74 | 9 |
| 24 | 33 | 10 |
| 24 | 43 | 36 |
| 25 | 33 | 10 |
| 26 | 297 | 11 |
| 27 | 216 | 12 |
| 28 | 181 | 12 |
| 28 | 186 | 51 |
| 28 | 253 | 35 |
| 29 | 271 | 13 |
| 30 | 118 | 14 |
| 31 | 258 | 15 |
| 32 | 194 | 16 |
| 32 | 278 | 30 |
| 33 | 184 | 18 |
| 34 | 137 | 26 |
| 34 | 203 | 18 |
| 35 | 250 | 27 |
| 35 | 304 | 42 |
| 35 | 305 | 18 |
| 35 | 319 | 51 |
| 36 | 245 | 22 |
| 36 | 245 | 37 |
| 36 | 269 | 20 |
| 36 | 317 | 6 |
| 36 | 338 | 18 |
| 36 | 340 | 56 |
| 37 | 189 | 19 |
| 38 | 172 | 2 |
| 38 | 303 | 20 |
| 39 | 289 | 20 |
| 40 | 289 | 20 |
| 41 | 273 | 20 |
| 42 | 328 | 20 |
| 43 | 296 | 21 |
| 44 | 315 | 22 |
| 45 | 271 | 2 |
| 45 | 312 | 40 |
| 46 | 151 | 23 |
| 47 | 103 | 39 |
| 47 | 130 | 25 |
| 48 | 178 | 26 |
| 49 | 196 | 31 |
| 49 | 226 | 26 |
| 49 | 226 | 61 |
| 50 | 294 | 27 |
| 51 | 278 | 42 |
| 51 | 295 | 27 |
| 51 | 305 | 35 |
| 52 | 295 | 27 |
| 53 | 295 | 27 |
| 53 | 295 | 35 |
| 54 | 294 | 59 |
| 54 | 295 | 27 |
| 55 | 36 | 29 |
| 56 | 226 | 51 |
| 56 | 271 | 2 |
| 56 | 271 | 8 |
| 57 | 118 | 53 |
| 57 | 186 | 29 |
| 58 | 121 | 62 |
| 58 | 167 | 52 |
| 58 | 181 | 29 |
| 59 | 250 | 35 |
| 59 | 278 | 30 |
| 60 | 245 | 31 |
| 60 | 245 | 40 |
| 61 | 277 | 31 |
| 62 | 221 | 34 |
| 63 | 217 | 34 |
| 64 | 207 | 25 |
| 64 | 259 | 35 |
| 64 | 321 | 42 |
| 65 | 272 | 3 |
| 66 | 330 | 37 |
| 67 | 277 | 37 |
| 68 | 100 | 38 |
| 69 | 104 | 39 |
| 70 | 186 | 40 |
| 71 | 335 | 40 |
| 72 | 253 | 42 |
| 73 | 253 | 42 |
| 74 | 253 | 42 |
| 75 | 253 | 42 |
| 76 | 278 | 42 |
| 77 | 288 | 42 |
| 78 | 160 | 44 |
| 79 | 191 | 44 |
| 80 | 312 | 2 |
| 81 | 191 | 44 |
| 82 | 186 | 45 |
| 83 | 229 | 45 |
| 84 | 226 | 45 |
| 85 | 267 | 46 |
| 86 | 246 | 51 |
| 86 | 262 | 46 |
| 87 | 191 | 49 |
| 87 | 191 | 55 |
| 87 | 191 | 56 |
| 87 | 191 | 56 |
| 88 | 118 | 53 |

**Total number of sampling occasions: 64*

**Table S6.** Sampling occasions during which each of the traps were active (i.e., sampled) for elephants in Kaziranga.

| Trap ID | Sampling Occasion |
| --- | --- |
| 1 | 5, 10, 16, 21, 28, 32, 34, 44, 49, 55, 58 |
| 2 | 5, 10, 16, 21, 28, 34, 44, 49, 55, 58 |
| 3 | 5, 10, 16, 21, 28, 34, 44, 49, 55, 58 |
| 4 | 5, 10, 16, 21, 28, 34, 44, 49, 55, 58 |
| 5 | 5, 10, 16, 21, 28, 34, 44, 49, 55, 58 |
| 6 | 5, 10, 16, 21, 28, 34, 44, 49, 55, 58 |
| 7 | 5, 10, 16, 21, 28, 34, 44, 49, 55, 58 |
| 8 | 5, 10, 16, 21, 28, 34, 44, 49, 55, 58 |
| 9 | 5, 10, 16, 21, 28, 34, 44, 49, 55, 58 |
| 10 | 5, 10, 16, 21, 28, 34, 44, 49, 55, 58 |
| 11 | 5, 10, 16, 21, 28, 34, 44, 49, 55, 58 |
| 12 | 5, 10, 16, 21, 28, 34, 44, 49, 55, 58 |
| 13 | 5, 10, 16, 21, 28, 34, 44, 49, 55, 58 |
| 14 | 5, 10, 16, 21, 28, 34, 44, 49, 55, 58 |
| 15 | 5, 10, 16, 21, 28, 34, 44, 49, 55, 58 |
| 16 | 5, 10, 16, 21, 28, 34, 44, 49, 55, 58 |
| 17 | 5, 10, 16, 21, 28, 34, 44, 49, 55, 58 |
| 18 | 5, 10, 16, 21, 28, 34, 44, 49, 55, 58 |
| 19 | 5, 10, 16, 21, 28, 34, 44, 49, 55, 58 |
| 20 | 5, 10, 16, 21, 28, 34, 44, 49, 55, 58 |
| 21 | 5, 10, 16, 21, 28, 34, 44, 49, 55, 58 |
| 22 | 5, 10, 16, 21, 28, 34, 44, 49, 55, 58 |
| 23 | 5, 10, 16, 21, 28, 34, 44, 49, 55, 58 |
| 24 | 5, 10, 16, 21, 28, 34, 44, 49, 55, 58 |
| 25 | 5, 10, 16, 21, 28, 34, 44, 49, 55, 58 |
| 26 | 5, 10, 16, 21, 28, 34, 44, 49, 55, 58 |
| 27 | 5, 10, 16, 21, 28, 34, 44, 49, 55, 58 |
| 28 | 5, 10, 16, 21, 28, 34, 44, 49, 55, 58 |
| 29 | 5, 10, 16, 21, 28, 34, 44, 49, 55, 58 |
| 30 | 5, 10, 16, 21, 28, 34, 44, 49, 55, 58 |
| 31 | 5, 10, 16, 21, 28, 34, 44, 49, 55, 58 |
| 32 | 5, 10, 16, 21, 28, 34, 44, 49, 55, 58 |
| 33 | 5, 10, 16, 21, 28, 34, 44, 49, 55, 58 |
| 34 | 5, 10, 16, 21, 28, 34, 44, 49, 55, 58 |
| 35 | 5, 10, 16, 21, 28, 34, 44, 49, 55, 58 |
| 36 | 7, 12, 17, 29, 36, 41, 47, 51, 57, 60 |
| 37 | 7, 12, 17, 29, 36, 41, 47, 51, 57, 60 |
| 38 | 7, 12, 17, 29, 36, 41, 47, 51, 57, 60 |
| 39 | 7, 12, 17, 29, 36, 41, 47, 51, 57, 60 |
| 40 | 7, 12, 17, 29, 36, 41, 47, 51, 57, 60 |
| 41 | 7, 12, 17, 29, 36, 41, 47, 51, 57, 60 |
| 42 | 7, 12, 17, 29, 36, 41, 47, 51, 57, 60 |
| 43 | 7, 12, 17, 29, 36, 41, 47, 51, 57, 60 |
| 44 | 7, 12, 17, 29, 36, 41, 47, 51, 57, 60 |
| 45 | 7, 12, 17, 29, 36, 41, 47, 51, 57, 60 |
| 46 | 7, 12, 17, 29, 36, 41, 47, 51, 57, 60 |
| 47 | 7, 12, 17, 29, 36, 41, 47, 51, 57, 60 |
| 48 | 7, 12, 17, 29, 36, 41, 47, 51, 57, 60 |
| 49 | 7, 12, 17, 29, 36, 41, 47, 51, 57, 60 |
| 50 | 7, 12, 17, 29, 36, 41, 47, 51, 57, 60 |
| 51 | 7, 12, 17, 29, 36, 41, 47, 51, 57, 60 |
| 52 | 7, 12, 17, 29, 36, 41, 47, 51, 57, 60 |
| 53 | 7, 12, 17, 29, 36, 41, 47, 51, 57, 60 |
| 54 | 7, 12, 17, 29, 36, 41, 47, 51, 57, 60 |
| 55 | 7, 12, 17, 29, 36, 41, 47, 51, 57, 60 |
| 56 | 7, 12, 17, 29, 36, 41, 47, 51, 57, 60 |
| 57 | 7, 12, 17, 29, 36, 41, 47, 51, 57, 60 |
| 58 | 7, 12, 17, 29, 36, 41, 47, 51, 57, 60 |
| 59 | 7, 12, 17, 29, 36, 41, 47, 51, 57, 60 |
| 60 | 7, 12, 17, 29, 36, 41, 47, 51, 57, 60 |
| 61 | 7, 12, 17, 29, 36, 41, 47, 51, 57, 60 |
| 62 | 7, 12, 17, 29, 36, 41, 47, 51, 57, 60 |
| 63 | 7, 12, 17, 29, 36, 41, 47, 51, 57, 60 |
| 64 | 7, 12, 17, 29, 36, 41, 47, 51, 57, 60 |
| 65 | 7, 12, 17, 29, 36, 41, 47, 51, 57, 60 |
| 66 | 3, 7, 9, 12, 14, 17, 19, 29, 32, 36, 38, 41, 45, 47, 51, 53, 57, 60, 62 |
| 67 | 3, 9, 14, 19, 32, 38, 45, 53, 62 |
| 68 | 3, 9, 14, 19, 32, 38, 45, 53, 62 |
| 69 | 3, 9, 14, 19, 32, 38, 45, 47, 53, 62 |
| 70 | 3, 9, 14, 19, 32, 38, 45, 53, 62 |
| 71 | 3, 9, 14, 19, 32, 38, 45, 53, 62 |
| 72 | 3, 9, 14, 19, 32, 38, 45, 53, 62 |
| 73 | 3, 9, 14, 19, 32, 38, 45, 53, 62 |
| 74 | 3, 9, 14, 19, 32, 38, 45, 53, 62 |
| 75 | 3, 9, 14, 19, 32, 38, 45, 53, 62 |
| 76 | 3, 9, 14, 19, 32, 38, 45, 53, 62 |
| 77 | 3, 9, 14, 19, 32, 38, 45, 53, 62 |
| 78 | 3, 9, 14, 19, 32, 38, 45, 53, 62 |
| 79 | 3, 9, 14, 19, 32, 38, 45, 53, 62 |
| 80 | 3, 9, 14, 19, 32, 38, 45, 53, 62 |
| 81 | 3, 9, 14, 19, 32, 38, 45, 53, 62 |
| 82 | 3, 9, 14, 19, 32, 38, 45, 53, 62 |
| 83 | 3, 9, 14, 19, 32, 38, 45, 53, 62 |
| 84 | 3, 9, 14, 19, 32, 38, 45, 53, 62 |
| 85 | 3, 9, 14, 19, 32, 38, 45, 53, 62 |
| 86 | 7, 12, 17, 29, 36, 41, 47, 51, 57, 60 |
| 87 | 7, 12, 17, 29, 36, 41, 47, 51, 57, 60 |
| 88 | 7, 12, 17, 29, 36, 41, 47, 51, 57, 60 |
| 89 | 7, 12, 17, 29, 36, 41, 47, 51, 57, 60 |
| 90 | 7, 12, 17, 29, 36, 41, 47, 51, 57, 60 |
| 91 | 7, 12, 17, 29, 36, 41, 47, 51, 57, 60 |
| 92 | 3, 9, 14, 19, 32, 38, 45, 53, 62 |
| 93 | 3, 9, 14, 19, 32, 38, 45, 53, 62 |
| 94 | 3, 9, 14, 19, 32, 38, 45, 53, 62 |
| 95 | 7, 12, 17, 29, 36, 41, 47, 51, 57, 60 |
| 96 | 7, 12, 17, 29, 32, 36, 41, 47, 51, 57, 60 |
| 97 | 7, 12, 17, 29, 32, 36, 41, 45, 47, 51, 53, 57, 60, 62 |
| 98 | 3, 9, 14, 19, 32, 38, 45, 53, 62 |
| 99 | 3, 9, 14, 19, 32, 38, 45, 53, 62 |
| 100 | 3, 9, 14, 19, 32, 38, 45, 53, 62 |
| 101 | 3, 9, 14, 19, 32, 38, 45, 53, 62 |
| 102 | 3, 4, 14, 16, 19, 25, 26, 31, 32, 39, 40, 44, 45, 48, 51, 54, 56, 58, 61 |
| 103 | 3, 4, 13, 14, 16, 19, 25, 26, 31, 32, 39, 40, 44, 45, 48, 51, 54, 56, 58, 61 |
| 104 | 3, 4, 13, 14, 16, 19, 25, 26, 31, 32, 39, 40, 44, 45, 48, 51, 54, 56, 58, 61 |
| 105 | 3, 4, 13, 14, 16, 19, 25, 26, 31, 32, 39, 40, 44, 45, 48, 51, 54, 56, 58, 61 |
| 106 | 4, 13, 19, 25, 32, 40, 45, 48, 54, 57 |
| 107 | 4, 13, 19, 25, 32, 40, 45, 48, 54, 57 |
| 108 | 4, 13, 19, 25, 32, 40, 45, 48, 54, 57 |
| 109 | 4, 13, 19, 25, 32, 40, 45, 48, 54, 57 |
| 110 | 4, 13, 19, 25, 32, 40, 45, 48, 54, 57 |
| 111 | 7, 9, 12, 14, 17, 19, 29, 32, 36, 38, 41, 45, 47, 51, 53, 58, 60, 62 |
| 112 | 7, 9, 12, 14, 17, 19, 29, 32, 36, 38, 41, 45, 47, 51, 53, 58, 60, 62 |
| 113 | 7, 9, 12, 14, 17, 19, 29, 32, 36, 38, 41, 45, 47, 51, 53, 58, 60, 62 |
| 114 | 3, 9, 14, 19, 32, 38, 45, 47, 51, 53, 62 |
| 115 | 3, 9, 14, 19, 32, 38, 45, 53, 62 |
| 116 | 3, 9, 14, 19, 32, 38, 45, 53, 62 |
| 117 | 3, 9, 14, 19, 32, 38, 45, 53, 62 |
| 118 | 3, 9, 14, 19, 32, 38, 45, 53, 62 |
| 119 | 3, 9, 14, 19, 32, 38, 45, 53, 62 |
| 120 | 3, 9, 14, 19, 32, 38, 45, 53, 62 |
| 121 | 3, 9, 14, 19, 32, 38, 45, 53, 62 |
| 122 | 3, 9, 14, 19, 32, 38, 45, 53, 62 |
| 123 | 3, 9, 14, 19, 32, 38, 45, 53, 62 |
| 124 | 3, 4, 13, 14, 16, 19, 25, 26, 31, 32, 39, 40, 44, 45, 48, 51, 54, 56, 58, 61 |
| 125 | 3, 4, 13, 14, 16, 19, 25, 26, 31, 32, 39, 40, 44, 45, 48, 51, 54, 56, 58, 61 |
| 126 | 3, 9, 14, 19, 32, 38, 45, 47, 53, 62 |
| 127 | 3, 9, 14, 19, 32, 38, 45, 47, 53, 62 |
| 128 | 3, 4, 13, 14, 16, 19, 25, 26, 31, 32, 39, 40, 44, 45, 48, 51, 54, 56, 58, 61 |
| 129 | 3, 4, 13, 14, 16, 19, 25, 26, 31, 32, 39, 40, 44, 45, 48, 51, 54, 56, 58, 61 |
| 130 | 3, 4, 13, 14, 16, 19, 25, 26, 31, 32, 39, 40, 44, 45, 48, 51, 54, 56, 58, 61 |
| 131 | 3, 4, 13, 14, 16, 19, 25, 26, 31, 32, 39, 40, 44, 45, 48, 51, 54, 56, 58, 61 |
| 132 | 3, 4, 13, 14, 16, 25, 26, 31, 32, 39, 40, 44, 45, 48, 51, 54, 56, 58, 61 |
| 133 | 3, 4, 13, 14, 16, 19, 25, 26, 31, 32, 39, 40, 44, 45, 48, 51, 54, 56, 58, 61 |
| 134 | 3, 4, 13, 14, 16, 19, 25, 26, 31, 32, 39, 40, 44, 45, 48, 51, 54, 56, 58, 61 |
| 135 | 3, 4, 13, 14, 16, 19, 25, 26, 31, 32, 39, 40, 44, 45, 48, 51, 54, 56, 58, 61 |
| 136 | 3, 4, 13, 14, 16, 19, 25, 26, 31, 32, 39, 40, 44, 45, 48, 51, 54, 56, 58, 61 |
| 137 | 3, 4, 13, 14, 16, 19, 25, 26, 31, 32, 39, 40, 44, 45, 48, 51, 54, 56, 58, 61 |
| 138 | 3, 4, 13, 14, 16, 19, 25, 26, 31, 32, 39, 40, 44, 45, 48, 51, 54, 56, 58, 61 |
| 139 | 3, 4, 13, 14, 16, 19, 25, 26, 31, 32, 39, 40, 44, 45, 48, 51, 54, 56, 58, 61 |
| 140 | 3, 4, 13, 14, 16, 19, 25, 26, 31, 32, 39, 40, 44, 45, 48, 51, 54, 56, 58, 61 |
| 141 | 3, 4, 13, 14, 16, 19, 25, 26, 31, 32, 39, 40, 44, 45, 48, 51, 54, 56, 58, 61 |
| 142 | 3, 4, 13, 14, 16, 19, 25, 26, 31, 32, 39, 40, 44, 45, 48, 51, 54, 56, 58, 61 |
| 143 | 3, 4, 13, 14, 16, 19, 25, 26, 31, 32, 39, 40, 44, 45, 48, 51, 54, 56, 58, 61 |
| 144 | 3, 4, 13, 14, 16, 19, 25, 26, 31, 32, 39, 40, 44, 45, 48, 51, 54, 56, 58, 61 |
| 145 | 3, 4, 13, 14, 16, 19, 25, 26, 31, 32, 39, 40, 44, 45, 48, 51, 54, 56, 58, 61 |
| 146 | 3, 4, 13, 14, 16, 19, 25, 26, 31, 32, 39, 40, 44, 45, 48, 51, 54, 56, 58, 61 |
| 147 | 3, 9, 14, 19, 32, 38, 45, 53, 62 |
| 148 | 2, 3, 9, 14, 16, 20, 23, 26, 28, 31, 33, 40, 41, 45, 47, 51, 52, 56, 61, 62 |
| 149 | 2, 9, 20, 23, 28, 33, 41, 47, 52, 62 |
| 150 | 2, 9, 20, 23, 28, 33, 41, 47, 52, 62 |
| 151 | 2, 9, 20, 23, 28, 33, 41, 47, 52, 62 |
| 152 | 2, 9, 20, 23, 28, 33, 41, 47, 52, 62 |
| 153 | 2, 9, 62 |
| 154 | 2, 9, 20, 23, 28, 33, 41, 47, 52, 62 |
| 155 | 2, 3, 9, 62 |
| 156 | 2, 9, 20, 23, 28, 33, 41, 47, 52, 62 |
| 157 | 2, 9, 20, 23, 28, 33, 41, 47, 52, 62 |
| 158 | 2, 9, 20, 23, 28, 33, 41, 47, 52, 62 |
| 159 | 2, 9, 20, 23, 28, 33, 41, 47, 52, 62 |
| 160 | 2, 9, 20, 23, 28, 33, 41, 44, 47, 52, 56, 62 |
| 161 | 2, 3, 4, 13, 14, 16, 19, 20, 23, 25, 26, 28, 31, 32, 33, 39, 40, 41, 44, 45, 47, 48, 51, 52, 54, 56, 58, 61, 62 |
| 162 | 2, 9, 20, 23, 28, 33, 41, 47, 52, 62 |
| 163 | 2, 9, 20, 23, 28, 33, 41, 47, 52, 62 |
| 164 | 2, 9, 20, 23, 28, 33, 41, 47, 52, 62 |
| 165 | 2, 9, 20, 23, 28, 33, 41, 47, 52, 62 |
| 166 | 2, 9, 20, 23, 28, 33, 41, 47, 52, 62 |
| 167 | 2, 9, 20, 23, 28, 33, 41, 47, 52, 62 |
| 168 | 3, 14, 16, 26, 31, 40, 45, 51, 56, 61 |
| 169 | 3, 14, 16, 26, 31, 40, 45, 51, 56, 61 |
| 170 | 3, 14, 16, 26, 31, 40, 45, 51, 56, 61 |
| 171 | 2, 8, 9, 20, 23, 28, 33, 41, 47, 52, 62 |
| 172 | 2, 9, 20, 23, 28, 33, 41, 47, 52, 62 |
| 173 | 3, 14, 16, 26, 31, 40, 45, 51, 56, 61 |
| 174 | 3, 14, 16, 26, 31, 40, 45, 51, 56, 61 |
| 175 | 2, 3, 4, 8, 9, 12, 13, 14, 16, 18, 19, 20, 23, 24, 25, 26, 28, 29, 31, 32, 33, 34, 39, 40, 41, 43, 44, 45, 47, 48, 49, 51, 52, 54, 55, 56, 58, 60, 61, 62 |
| 176 | 2, 4, 9, 13, 19, 20, 23, 25, 28, 32, 33, 39, 41, 44, 47, 48, 52, 54, 58, 62 |
| 177 | 3, 14, 16, 26, 31, 40, 45, 51, 56, 61 |
| 178 | 3, 14, 16, 26, 31, 40, 45, 51, 56, 61 |
| 179 | 3, 14, 16, 26, 31, 40, 45, 51, 56, 61 |
| 180 | 2, 8, 9, 12, 18, 20, 23, 24, 28, 29, 33, 34, 41, 43, 47, 49, 52, 55, 60, 62 |
| 181 | 2, 8, 9, 12, 18, 20, 23, 24, 28, 29, 33, 34, 41, 43, 47, 49, 52, 55, 60, 62 |
| 182 | 8, 12, 18, 24, 29, 34, 43, 49, 55, 60 |
| 183 | 8, 12, 18, 24, 29, 34, 43, 49, 55, 60 |
| 184 | 2, 3, 4, 8, 9, 12, 13, 14, 16, 18, 19, 20, 23, 24, 25, 26, 28, 29, 31, 32, 33, 34, 39, 40, 41, 43, 44, 45, 47, 48, 49, 51, 52, 54, 55, 56, 58, 60, 61, 62 |
| 185 | 8, 12, 18, 24, 29, 34, 43, 49, 55, 60 |
| 186 | 2, 3, 4, 8, 9, 12, 13, 14, 16, 18, 19, 20, 23, 24, 25, 26, 28, 29, 31, 32, 33, 34, 39, 40, 41, 43, 44, 45, 47, 48, 49, 51, 52, 54, 55, 56, 58, 60, 61, 62 |
| 187 | 8, 12, 18, 24, 29, 34, 43, 49, 55, 60 |
| 188 | 2, 4, 8, 9, 12, 13, 14, 16, 18, 19, 20, 23, 24, 25, 26, 28, 29, 31, 32, 33, 34, 39, 40, 41, 43, 44, 45, 47, 48, 49, 51, 52, 54, 55, 56, 58, 60, 61, 62 |
| 189 | 2, 3, 4, 8, 9, 12, 13, 14, 16, 18, 19, 20, 23, 24, 25, 26, 28, 29, 31, 32, 33, 34, 39, 40, 41, 43, 44, 45, 47, 48, 49, 51, 52, 54, 55, 56, 58, 60, 61, 62 |
| 190 | 2, 3, 4, 8, 9, 12, 13, 14, 16, 18, 19, 20, 23, 24, 25, 26, 28, 29, 31, 32, 33, 34, 39, 40, 41, 43, 44, 45, 47, 48, 49, 51, 52, 54, 55, 56, 58, 60, 61, 62 |
| 191 | 2, 3, 4, 8, 9, 12, 13, 14, 16, 18, 19, 20, 23, 24, 25, 26, 28, 29, 31, 32, 33, 34, 39, 40, 41, 43, 44, 45, 47, 48, 49, 51, 52, 54, 55, 56, 58, 60, 61, 62 |
| 192 | 3, 14, 16, 26, 31, 40, 45, 51, 56, 61 |
| 193 | 3, 14, 16, 26, 31, 40, 45, 51, 56, 61 |
| 194 | 3, 14, 16, 26, 31, 40, 45, 51, 56, 61 |
| 195 | 2, 8, 9, 12, 18, 20, 23, 24, 28, 29, 33, 34, 41, 43, 47, 49, 52, 55, 60, 62 |
| 196 | 3, 14, 16, 26, 31, 40, 45, 51, 56, 61 |
| 197 | 3, 14, 16, 26, 31, 40, 45, 51, 56, 61 |
| 198 | 3, 14, 16, 26, 31, 40, 45, 51, 56, 61 |
| 199 | 3, 14, 16, 26, 31, 40, 45, 51, 56, 61 |
| 200 | 3, 14, 16, 26, 31, 40, 45, 51, 56, 61 |
| 201 | 8, 12, 18, 21, 24, 29, 34, 35, 42, 43, 46, 49, 55, 59, 60, 64 |
| 202 | 8, 12, 18, 24, 29, 34, 43, 49, 55, 60 |
| 203 | 8, 12, 18, 24, 29, 34, 43, 49, 55, 60 |
| 204 | 8, 12, 18, 24, 29, 34, 43, 49, 55, 60 |
| 205 | 8, 12, 18, 24, 29, 34, 43, 49, 55, 60 |
| 206 | 8, 12, 18, 24, 29, 34, 43, 49, 55, 60 |
| 207 | 8, 12, 18, 24, 25, 29, 34, 43, 49, 55, 60 |
| 208 | 8, 12, 18, 24, 29, 34, 43, 49, 55, 60 |
| 209 | 8, 12, 18, 24, 29, 34, 43, 49, 55, 60 |
| 210 | 8, 12, 18, 24, 29, 34, 43, 49, 55, 60 |
| 211 | 8, 12, 18, 24, 29, 34, 43, 49, 55, 60 |
| 212 | 8, 12, 18, 24, 29, 34, 43, 49, 55, 60 |
| 213 | 8, 12, 18, 24, 29, 34, 43, 49, 55, 60 |
| 214 | 8, 12, 18, 24, 29, 34, 43, 49, 55, 60 |
| 215 | 8, 12, 18, 24, 29, 34, 43, 49, 55, 60 |
| 216 | 8, 12, 18, 24, 29, 34, 43, 49, 55, 60 |
| 217 | 8, 12, 18, 24, 29, 34, 43, 49, 55, 60 |
| 218 | 3, 14, 26, 31, 40, 45, 51, 56, 61 |
| 219 | 3, 14, 16, 26, 31, 40, 45, 51, 56, 61 |
| 220 | 3, 14, 16, 26, 31, 40, 45, 51, 56, 61 |
| 221 | 8, 12, 18, 24, 29, 34, 43, 49, 55, 60 |
| 222 | 8, 12, 18, 24, 29, 34, 43, 49, 55, 60 |
| 223 | 5, 8, 11, 12, 15, 18, 21, 24, 27, 29, 34, 35, 42, 43, 46, 49, 55, 59, 60, 64 |
| 224 | 5, 8, 11, 12, 15, 18, 21, 24, 27, 29, 34, 35, 42, 43, 46, 49, 55, 59, 60, 64 |
| 225 | 5, 8, 11, 12, 15, 18, 21, 24, 27, 29, 34, 35, 42, 43, 46, 49, 55, 59, 60, 64 |
| 226 | 3, 14, 16, 26, 31, 40, 45, 51, 56, 61 |
| 227 | 3, 14, 16, 26, 31, 40, 45, 51, 56, 61 |
| 228 | 3, 14, 16, 26, 31, 40, 45, 51, 56, 61 |
| 229 | 3, 14, 16, 26, 31, 40, 45, 51, 56, 61 |
| 230 | 3, 14, 16, 26, 31, 40, 45, 51, 56, 61 |
| 231 | 3, 14, 16, 26, 31, 40, 45, 51, 56, 61 |
| 232 | 3, 14, 16, 26, 31, 40, 45, 51, 56, 61 |
| 233 | 3, 14, 16, 26, 31, 40, 45, 51, 56, 61 |
| 234 | 3, 14, 16, 26, 31, 40, 45, 51, 56, 61 |
| 235 | 5, 11, 15, 21, 27, 35, 42, 46, 59, 64 |
| 236 | 5, 11, 15, 21, 27, 35, 42, 46, 59, 64 |
| 237 | 5, 11, 21, 27, 35, 42, 46, 59, 64 |
| 238 | 3, 14, 16, 26, 31, 40, 45, 51, 56, 61 |
| 239 | 3, 14, 16, 26, 31, 40, 45, 51, 56, 61 |
| 240 | 8, 12, 18, 24, 29, 34, 43, 49, 55, 60 |
| 241 | 8, 12, 18, 24, 29, 34, 43, 49, 55, 60 |
| 242 | 5, 11, 15, 21, 27, 35, 42, 46, 59, 64 |
| 243 | 1, 2, 3, 6, 8, 13, 18, 20, 22, 26, 31, 37, 40, 45, 46, 51, 52, 56, 63 |
| 244 | 1, 2, 6, 8, 13, 18, 20, 22, 30, 31, 37, 40, 46, 52, 63 |
| 245 | 1, 2, 6, 8, 13, 18, 20, 22, 30, 31, 37, 40, 46, 52, 63 |
| 246 | 1, 2, 6, 8, 13, 18, 20, 22, 30, 31, 37, 40, 46, 51, 52, 63 |
| 247 | 5, 11, 15, 21, 27, 35, 42, 46, 59, 64 |
| 248 | 5, 11, 15, 21, 27, 35, 42, 46, 59, 64 |
| 249 | 5, 11, 15, 21, 27, 35, 42, 46, 59, 64 |
| 250 | 5, 11, 15, 21, 27, 35, 42, 46, 59, 64 |
| 251 | 5, 11, 15, 21, 27, 35, 42, 46, 59, 64 |
| 252 | 5, 11, 15, 21, 27, 35, 42, 46, 59, 64 |
| 253 | 5, 11, 15, 21, 27, 35, 42, 46, 59, 64 |
| 254 | 5, 8, 11, 12, 15, 18, 21, 24, 27, 29, 34, 35, 42, 43, 46, 49, 55, 59, 60, 64 |
| 255 | 5, 8, 11, 12, 15, 18, 21, 24, 27, 29, 34, 35, 42, 43, 46, 49, 55, 59, 60, 64 |
| 256 | 5, 8, 11, 12, 15, 18, 21, 24, 27, 29, 34, 35, 42, 43, 46, 49, 55, 59, 60, 64 |
| 257 | 5, 11, 15, 21, 27, 35, 42, 46, 59, 64 |
| 258 | 5, 11, 15, 21, 27, 35, 42, 46, 59, 64 |
| 259 | 1, 2, 6, 8, 13, 18, 20, 22, 30, 31, 35, 37, 40, 42, 46, 50, 52, 56, 59, 63 |
| 260 | 1, 2, 6, 8, 13, 18, 20, 22, 30, 37, 40, 46, 52, 63 |
| 261 | 1, 2, 6, 8, 13, 18, 20, 22, 30, 31, 35, 37, 40, 46, 50, 52, 56, 59, 63 |
| 262 | 1, 2, 6, 8, 13, 18, 20, 30, 31, 37, 40, 46, 52, 63 |
| 263 | 1, 2, 6, 8, 13, 18, 20, 22, 30, 31, 35, 37, 40, 42, 46, 50, 52, 56, 59, 63 |
| 264 | 1, 2, 6, 8, 13, 18, 20, 22, 30, 31, 35, 37, 40, 42, 46, 50, 52, 56, 59, 63 |
| 265 | 5, 11, 15, 21, 27, 35, 42, 46, 59, 64 |
| 266 | 5, 11, 15, 21, 27, 35, 42, 46, 59, 64 |
| 267 | 5, 11, 15, 21, 27, 35, 42, 46, 59, 64 |
| 268 | 1, 8, 18, 20, 30, 35, 42, 46, 50, 56, 59 |
| 269 | 1, 8, 18, 20, 30, 35, 42, 50, 56, 59 |
| 270 | 1, 2, 6, 8, 13, 18, 20, 22, 30, 31, 35, 37, 40, 42, 46, 50, 52, 56, 59, 63 |
| 271 | 1, 2, 6, 8, 13, 18, 20, 22, 30, 31, 35, 37, 40, 42, 46, 50, 52, 56, 59, 63 |
| 272 | 1, 2, 3, 6, 8, 13, 18, 20, 22, 30, 31, 35, 37, 40, 42, 46, 50, 52, 56, 59, 63 |
| 273 | 1, 2, 3, 6, 8, 13, 18, 20, 22, 30, 31, 35, 37, 40, 42, 46, 50, 52, 56, 59, 63 |
| 274 | 1, 2, 3, 6, 8, 13, 18, 20, 22, 30, 31, 35, 37, 40, 42, 46, 50, 52, 56, 59, 63 |
| 275 | 2, 6, 13, 22, 31, 37, 40, 46, 52, 63 |
| 276 | 2, 6, 13, 22, 31, 37, 40, 46, 52, 63 |
| 277 | 2, 6, 13, 22, 31, 37, 40, 46, 52, 63 |
| 278 | 1, 8, 18, 20, 30, 35, 42, 50, 56, 59 |
| 279 | 1, 8, 18, 20, 30, 35, 42, 50, 56, 59 |
| 280 | 1, 8, 18, 20, 30, 35, 42, 50, 56, 59 |
| 281 | 1, 8, 18, 20, 30, 35, 42, 50, 56, 59 |
| 282 | 1, 8, 18, 20, 30, 35, 42, 50, 56, 59 |
| 283 | 1, 8, 18, 20, 30, 35, 42, 50, 56, 59 |
| 284 | 1, 8, 18, 20, 30, 35, 42, 50, 56, 59 |
| 285 | 1, 8, 18, 20, 30, 35, 42, 50, 56, 59 |
| 286 | 1, 8, 18, 20, 30, 35, 42, 50, 56, 59 |
| 287 | 1, 8, 18, 20, 30, 35, 42, 50, 56, 59 |
| 288 | 1, 8, 18, 20, 30, 35, 42, 50, 56, 59 |
| 289 | 1, 8, 18, 20, 30, 35, 42, 50, 56, 59 |
| 290 | 1, 8, 18, 20, 30, 35, 42, 50, 56, 59 |
| 291 | 1, 8, 18, 20, 30, 35, 42, 50, 56, 59 |
| 292 | 1, 8, 18, 20, 30, 35, 42, 50, 56, 59 |
| 293 | 1, 8, 18, 20, 30, 35, 42, 50, 56, 59 |
| 294 | 5, 11, 15, 21, 27, 35, 42, 46, 59, 64 |
| 295 | 5, 11, 15, 21, 27, 35, 42, 46, 59, 64 |
| 296 | 5, 11, 15, 21, 27, 35, 42, 46, 59, 64 |
| 297 | 5, 11, 15, 21, 27, 35, 42, 46, 59, 64 |
| 298 | 5, 11, 15, 21, 27, 35, 42, 46, 59, 64 |
| 299 | 5, 11, 15, 21, 27, 35, 42, 46, 59, 64 |
| 300 | 11, 35 |
| 301 | 1, 8, 18, 20, 30, 35, 42, 50, 56, 59 |
| 302 | 1, 8, 18, 20, 30, 35, 42, 50, 56, 59 |
| 303 | 1, 8, 18, 20, 30, 35, 42, 50, 56, 59 |
| 304 | 1, 8, 18, 20, 30, 35, 42, 50, 56, 59 |
| 305 | 1, 8, 18, 20, 30, 35, 42, 50, 56, 59 |
| 306 | 1, 8, 18, 20, 30, 35, 42, 50, 56, 59 |
| 307 | 5, 11, 15, 21, 27, 35, 42, 46, 59, 64 |
| 308 | 5, 11, 15, 21, 27, 35, 42, 46, 59, 64 |
| 309 | 5, 11, 15, 21, 27, 35, 42, 46, 59, 64 |
| 310 | 5, 11, 15, 21, 27, 35, 42, 46, 59, 64 |
| 311 | 2, 6, 13, 22, 31, 37, 40, 46, 52, 63 |
| 312 | 2, 6, 13, 22, 31, 37, 40, 46, 52, 63 |
| 313 | 2, 6, 13, 22, 31, 37, 40, 46, 52, 63 |
| 314 | 2, 6, 13, 22, 31, 37, 40, 46, 52, 63 |
| 315 | 2, 6, 13, 22, 31, 37, 40, 46, 52, 63 |
| 316 | 2, 6, 13, 22, 31, 37, 40, 46, 52, 63 |
| 317 | 1, 2, 6, 8, 13, 18, 20, 22, 30, 31, 35, 37, 40, 42, 46, 50, 52, 56, 59, 63 |
| 318 | 1, 2, 6, 8, 13, 18, 20, 22, 30, 31, 35, 37, 40, 42, 46, 50, 52, 56, 59, 63 |
| 319 | 1, 2, 6, 8, 13, 18, 20, 22, 30, 31, 35, 37, 40, 42, 46, 50, 52, 56, 59, 63 |
| 320 | 1, 2, 6, 8, 13, 18, 20, 22, 30, 31, 35, 37, 40, 42, 46, 50, 52, 56, 59, 63 |
| 321 | 1, 2, 6, 8, 13, 18, 20, 22, 30, 31, 35, 37, 40, 42, 46, 50, 52, 56, 59, 63 |
| 322 | 1, 8, 18, 20, 30, 35, 42, 50, 56, 59 |
| 323 | 1, 8, 18, 20, 30, 35, 42, 50, 56, 59 |
| 324 | 1, 8, 18, 20, 30, 35, 42, 50, 56, 59 |
| 325 | 1, 8, 18, 20, 30, 35, 42, 50, 56, 59 |
| 326 | 1, 8, 18, 20, 30, 35, 42, 50, 56, 59 |
| 327 | 1, 8, 18, 20, 30, 35, 42, 50, 56, 59 |
| 328 | 1, 8, 18, 20, 30, 35, 42, 50, 56, 59 |
| 329 | 2, 6, 13, 22, 31, 37, 40, 46, 52, 63 |
| 330 | 2, 6, 13, 22, 31, 37, 40, 46, 52, 63 |
| 331 | 2, 6, 13, 22, 31, 37, 40, 46, 52, 63 |
| 332 | 2, 6, 13, 22, 31, 37, 40, 46, 52, 63 |
| 333 | 2, 6, 13, 22, 31, 37, 40, 46, 52, 63 |
| 334 | 2, 6, 13, 22, 31, 37, 40, 46, 52, 63 |
| 335 | 2, 6, 13, 22, 31, 37, 40, 46, 52, 63 |
| 336 | 1, 8, 18, 20, 30, 35, 42, 50, 56, 59 |
| 337 | 1, 8, 18, 20, 30, 35, 42, 50, 56, 59 |
| 338 | 1, 8, 18, 20, 30, 35, 42, 50, 56, 59 |
| 339 | 1, 6, 8, 13, 18, 19, 22, 30, 31, 35, 37, 40, 41, 46, 50, 52, 56, 59, 63 |
| 340 | 1, 2, 6, 8, 13, 18, 19, 22, 30, 31, 35, 37, 41, 46, 50, 52, 56, 59, 63 |
| 341 | 1, 6, 8, 13, 18, 19, 22, 30, 31, 35, 37, 40, 41, 46, 50, 52, 56, 59, 63 |
| 342 | 2, 6, 13, 22, 31, 37, 40, 46, 52, 63 |
| 343 | 2, 6, 13, 22, 31, 37, 40, 46, 52, 63 |
| 344 | 2, 6, 13, 22, 31, 37, 40, 46, 52, 63 |
| 345 | 2, 6, 13, 22, 31, 37, 40, 46, 52, 63 |
| 346 | 2, 6, 13, 22, 31, 37, 40, 46, 52, 63 |
| 347 | 2, 6, 13, 22, 31, 37, 40, 46, 52, 63 |
| 348 | 1, 2, 6, 8, 13, 18, 20, 22, 30, 31, 35, 37, 40, 42, 46, 50, 52, 56, 59, 63 |
| 349 | 1, 2, 6, 8, 13, 18, 20, 22, 30, 31, 35, 37, 40, 42, 46, 50, 52, 56, 59, 63 |
| 350 | 2, 6, 13, 22, 31, 37, 40, 46, 52, 63 |
